# Precarious Hope: Situated Perspectives on the COVID-19 Pandemic from Undergraduate Students in Manchester, UK

**DOI:** 10.1007/s43151-021-00057-1

**Published:** 2021-11-12

**Authors:** Caitlin Nunn, Chloe Germaine, Charlotte Ogden, Yasmin Miah, Jessica Marsh, Rhiannon Kitching, Nasira Kathrada, Katerina Hough, Isabel Harper

**Affiliations:** 1 Manchester Centre for Youth Studies (MCYS), Manchester Metropolitan University, Manchester, UK; 2 Manchester Metropolitan University, Manchester, UK

**Keywords:** Youth, COVID-19, Higher education, Hope, Precarity, Co-production

## Abstract

The COVID-19 pandemic has had a profound impact on the lives of young people, transforming and disrupting education provision, employment opportunities, social practices, mobilities, and experiences of health and well-being. In the UK context, the pandemic can be understood as both a unique event and as a further addition to the intersecting crises—including austerity and Brexit—that are increasingly shaping and constraining youth experiences and aspirations and exacerbating precarity and inequality. In this article, seven undergraduate students from Manchester, UK, with two academic co-authors, employ a co-productive approach to reflect on our experiences of the pandemic. Our autoethnographic accounts draw attention to the situated effects of the pandemic, and its intersection with existing challenges and pressures, including the gig economy, mental and physical ill health, and transnational family networks. At the same time, our narratives capture a sense of *precarious hope*: hopefulness that is both a product of precarity and itself precarious, opening up new possibilities for collectively imagining and pursuing viable and meaningful futures in uncertain times. Supporting our endeavours requires the inclusion of youth voices in research, policy, and practice; work we begin here.

## Introduction

Since it was first identified in late 2019, COVID-19 has quickly and radically transformed life globally. While largely spared the worst health effects of the virus to date, young people have been disproportionately affected by the policies and practices implemented by governments to limit its spread. In the UK, where the pandemic has been particularly severe, this has included dramatic changes to education, high rates of unemployment, and ruptures in key life-stage transitions, social relations, and leisure practices, with concomitant effects on mental health and well-being (Prince’s Trust 2021; Gabriel et al. 2021). While this constellation of challenges is unique to the COVID-19 crisis, many issues were pre-existing, rendered visible or further exacerbated by the pandemic. In this context, the COVID-19 pandemic is yet another strand in the intersecting crises already shaping, constraining, and unsettling youth trajectories and futures.

As has been extensively evidenced in pre-pandemic youth studies research, young people’s lives were already precarious (e.g. Macdonald and Giazitzoglu 2019; Furlong et al 2018). Indeed, as Anna Tsing writes, ‘precarity *is* the condition of our time’ (2015: 20, emphasis in original). While precarity is primarily conceptualised in relation to the labour market, we argue that it transcends this context to characterise a wide range of contemporary relations. Globally, this includes our relationship with the natural world as we confront the climate crisis, while in the UK, ‘hostile environment’ migration policies, Brexit, and Black Lives Matter have heightened the perpetual precariousness of citizenship and belonging for racialised and migrant-background groups. Moreover, the generalised sense of instability and uncertainty that marks youth trajectories renders precarious the mental health of many young people (Milner et al. 2019). At the same time, young people, who will live with the legacies of and inherit the responsibility for these intersecting crises, are dramatically under-represented in public discussions of their impacts and the strategies required to address them (Gabriel et al. 2021).

In this article, seven undergraduate students studying in Manchester, UK, share our autoethnographic reflections on living through the pandemic. Our accounts, while reflecting diverse positionalities and experiences, share a profound sense of precarity: as a pre-existing condition, a result of the pandemic, and a force shaping possible futures. At the same time, these experiences of precarity are also shown to be generative, giving rise to hopefulness about the future: a sense that conditions should and could change. The pandemic has made apparent precarious lives, and, indeed, made them more so, but it has also created opportunities to discuss those conditions of precarity and the unequal ways in which they are experienced, including across race, gender, and class. These discussions are opening up new forms of recognition and solidarity among young people and a concomitant sense of possibilities for transformation inherent in conditions of precarity.

### Youth in the Time of COVID

In December 2019, the first case of COVID-19 was reported in Wuhan, China. By early 2020, the virus had spread across the globe to the extent that, by March, in the face of almost one thousand deaths, the UK government announced a national lockdown. This was to be followed by almost sixteen months of rolling restrictions and lockdowns as new waves hit, affecting every aspect of daily life. North West England, where this research is based, has been particularly badly affected, with Greater Manchester’s mortality from COVID-19 25% higher than England overall (Marmot et al 2021). Eighteen months since the virus was first reported in the UK, research on youth experiences of the pandemic is still emerging. However, surveys conducted by the Prince’s Trust, Centre for Economic Performance and Beatfreeks in 2020/2021 demonstrate the profound effects that the pandemic and associated lockdowns and restrictions have had on young people. Indeed, the Prince’s Trust (2021:8) reports that ‘scores for emotional health and work and education have reached their lowest point in Youth Index history’.

University students have been thrust into an online learning environment. The shift has rendered visible existing educational inequalities, including in relation to digital access and literacy; skills in self-directed learning; access to social, emotional, and practical support; and the nature of home environments, including space, relations, and responsibilities. These inequalities particularly affect first-generation students and those from disadvantaged and minoritised backgrounds (Literat 2021; Major et al 2020). At the same time, academic staff—themselves navigating new modes of delivery and wider pandemic-related personal and professional challenges—have had decreased capacity to support students through this transition (Burns et al 2020).

The pandemic has additionally exacerbated existing challenges associated with the transition to independent living that, for many students, comes with entering university. Many students have faced increased financial pressures due to a shrinking job market, and all have been denied the social life that is both a key aspect of the student experience and an important source of connection and support. International students and young people who have family members overseas have also faced the difficult predicament of whether to—or being unable to—travel, as well as anxiety about the health of family overseas and their constrained ability to give or receive care (Burns et al. 2021). More generally, young people report significant declines in mental health since the start of the pandemic, including increased anxiety and reduced ability to cope with life (Prince’s Trust 2021; Beatfreeks 2020). A study by Dewa et al. (2021) found that Black and Black British young people have been particularly affected and suggests that heightened anxiety among young people will continue into the future.

Changes in the labour market have disproportionately impacted young people, who are significantly more likely than older adults to have experienced job loss and to be unemployed. Young people already face higher under/unemployment rates and are overrepresented in industries curtailed by public health measures, such as hospitality and retail. They are also more likely to be precariously employed and without access to formal entitlements and protections (Macdonald and Giazitzoglu 2019). In this context, Cook et al. (2021:15) suggest that the pandemic presents ‘a crisis of precarious work itself’. Moreover, the pandemic has eroded young people’s confidence about future work and careers and increased uncertainty among those undertaking key transitions, such as completing education (Cook et al 2021; Prince’s Trust 2021; Major et al 2020; Beatfreeks 2020).

Throughout the pandemic, young people have been subject to high levels of social control through adultist (also raced, classed) policy, policing, and media reporting (Gabriel et al 2021), and have, more generally, not felt listened to. Analysing TikTok videos about home learning, Literat (2021:11) found that what emerged most strongly was ‘a feeling of powerlessness among students during this traumatic time…a sense of being talked *about* and decided *for*’. Yet at the same time, many young people are responding to the pandemic and associated challenges with an increased commitment to social responsibility and supporting those in need, and many young people retain a strong belief that ‘my generation can change our future for the better’ (Prince’s Trust 2021: 15). Drawing on pre-pandemic youth studies research, we might understand these hopeful acts and beliefs as a pragmatic strategy for dealing with uncertainty (Cook 2018), a source of personal satisfaction and happiness in the context of prevailing negativity and frustration (Bishop and Willis 2014), and a form of agency in the face of disempowerment (Bryant and Ellard 2015).

## Approach

This article began as a seminar hosted by Manchester Centre for Youth Studies (MCYS) at Manchester Metropolitan University. The seminar focused on intersecting crises in contemporary youth experiences and what they mean for university students as they prepare for their post-education futures. The seminar was co-produced with undergraduate students from across the Faculty of Arts and Humanities who volunteered to participate and were remunerated for their contribution. Emerging clearly through the seminar were the many ways in which the pandemic not only created new challenges—and at times opportunities—but also intersected with existing inequalities to amplify the current precarity and future uncertainty for young people. Also emerging strongly was the desire of students to speak about their experiences, frustration at the lack of avenues for communication, and gratitude for the opportunity to address the academic community. This served as the impetus for this article, co-authored by the academics and students who co-presented the seminar.

In this article, we bring together co-production and autoethnographic approaches to explore students’ experiences of the pandemic. In so doing, we draw on co-production’s ethos of centring the knowledge and understandings of non-academic co-researchers and autoethnography’s use of individual stories to illuminate broader sociocultural issues (Bell & Pahl 2018; Ellis et al., 2010). Co-production describes a range of approaches that facilitate collaboration among academic and non-academic researchers in the design, implementation, and dissemination of research. Such approaches aspire to realise less hierarchical relations and more democratic forms of knowledge production within and beyond the research project (Bell & Pahl 2018; Lenette et al 2019). Co-production is an apt approach for researching student experiences of the pandemic, explicitly addressing the underrepresentation of youth voices. Individual student experiences are explored in the article through autoethnographic vignettes. Both a mode of inquiry and of representation (Richardson 2000), autoethnography offers a unique insight into the lives of student authors: not only our ideas and experiences, but also our ways of knowing and modes of articulation (Ellis et al., 2010).

The seven student authors of this article had, at the time of writing, recently completed our second or third year of undergraduate degrees in English, Linguistics, and Sociology and were aged between twenty and twenty-two years. Reflecting the diversity of the student body, we come from a range of ethnic, national, and socioeconomic backgrounds, and several of us are first generation students. The two academic authors are staff members of MCYS from English and Sociology. The ideas collaboratively developed in the seminar were further developed for this article through online reflective discussions, autoethnographic writing, and peer feedback. Autoethnographic vignettes were co-analysed by academic and student authors, with a focus on identifying pre-existing and new challenges, emotional and practical responses, and perceived legacies.

Reflecting the hopeful orientation of student authors, ours is a hopeful approach. Co-production aspires to transformative change through disrupting privileged forms of academic knowledge production, modelling alternative approaches, and equipping community members with the confidence and resources to take action. Yet, it is a hope ‘surrounded by dangers’ (Bloch 1988, quoted in Bell & Pahl 2018: 108); that risks being co-opted by neoliberal impact and participation agendas within and beyond the academy. It is critical that we remain alert to individual and institutional practices that instrumentalise and tokenise co-production for financial and reputational gain, and that, in doing so, reinforce rather than dismantle hierarchies of knowledge and power. In this context, we endeavoured to approach our collaboration honestly, critically, and care-fully. Central to this was practicing reflexivity not only in our autoethnographic writing but also in our relations with each other, recognising our differing intersectional positions, and particularly mindful of—and seeking to mitigate—the power imbalances inherent in academic-student relations. We sought to create a cross-generational partnership that encouraged mutual learning: educating academic authors about student experiences and equipping student authors with transferrable skills and recognition to support their entry into an uncertain labour market.

## Youth Perspectives

The vignettes below focus on a range of aspects of our experiences during the pandemic, reflecting our situated perspectives. These include education and (un)employment, the complexities of transnational lives, and managing physical and mental health. Each vignette has a particular focus with respect to these challenges, though we recognise the intersectional nature of the crises we have navigated during the pandemic, as women, as women of colour, as students from disadvantaged backgrounds, as students with disabilities, and so on (Crenshaw 1991). We occupy complex locations within these crises, and our reflections surface the unique intersections therein.

### Rhiannon

I applied to university in 2017. As the first person in my family to do so, I was under pressure, largely from myself but everyone around me too, to succeed. I take some issue with the term ‘first generation student’ because it often carries stereotypes. I was aware of the low expectations people have for children who grow up in Barnsley, or areas like this. I say ‘areas like this’ to mean social deprivation, joblessness, low academic outcomes, but the phrase shows the extent to which we internalise stereotypes often without thinking about the human at the other end. There is a very real discourse on how my socio-economic position is likely to determine my academic and professional success, that suggests my postcode is an indicator of my academic outcomes. This was expressed by other parents at primary school, who told my Mum that I was less likely to succeed because she was a single parent, and in people’s visible shock when I got good SATs results, good GCSEs or good A-levels. Imposter syndrome has followed me everywhere.

We have unequal access to opportunities in the UK. The handling of the GCSE and A-Level grades last year is an important example. The failure of our governing bodies to accurately represent young people’s grades and to safeguard their futures was the pinnacle of this. To see prejudices about young people’s race and economic background reflected in the estimated grades was unforgivable. As soon as someone is bracketed – as I would’ve been if I was a GCSE student in 2020 – then we are automatically capping aspiration.

COVID-19 has exacerbated these feelings in some ways, but equally, spurred me on to defy expectations. The issues discussed above are a driving force for my desire to become a teacher. I feel incredibly privileged to have been accepted onto my first-choice teacher training programme commencing this September, in a time of incredible job instability, but I also feel that they need students like me more than ever. From the digital accessibility gap, brought to attention due to COVID, to the widening attainment gap, young people need teachers who can empathise with their circumstances. I feel I can do just that. COVID-19 has taught us a lot about empathy.

At this point, nearly 21 years too late, I have gained the belief of those onlookers from primary school. They’ve accepted I can do it. This is proof that the narratives we hear, those amplified during COVID-19, about deprived children and the learning gap, are harmful and are internalised. Collectively, I hope, we can tackle these issues.

### Isabel

I cannot afford this pandemic.

From the age of sixteen, I’ve always had a job: a babysitter, hotel receptionist, retail assistant. At eighteen, I moved into the hospitality sector and started waiting tables and pouring pints whilst I revised Shakespeare on my lunch break.

Being a first-generation student from a working-class Caribbean family meant that working whilst studying was a requirement, rather than a choice. Most young people take on some paid work whilst they study, as student finance doesn’t cover the costs of rent and course materials. Yet almost two-thirds of people who lost jobs in the UK pandemic were under 25 (ONS 2021). When the UK entered its first lockdown, I was already indefinitely unemployed. My previous contract ended on January 3^rd^, 2020, and I reluctantly boarded the merry-go-round of minimum wage-paying, social-life-preserving job hunting. Trying to find flexible part-time work in an over-competitive, over-saturated, capitalist job market is mentally and physically exhausting.

COVID-19 has depleted my motivation to compete for part-time employment. I’ve been through all the stages of grief: denial that we’d be in lockdown for too long, anger that the beginning of my twenties was happening in a global pandemic, bargaining that the infection and death rates would decrease enough in the summer, depression from the ridiculous influx of rejected job applications and lack of emotional support, and acceptance that the pandemic is in no rush of disappearing. Unemployment contributed to the decline of my mental health. When I had to borrow money from my family, I felt like I was regressing. I chose to go to university to learn how to become an adult, and yet, at twenty-one years old, I’ve never felt more like a child.

When the expectation for your demographic is to find work, but the world has shut down and there is no new work available, how are young people supposed to react? Conversations around mental health have been prevalent in the media since the pandemic started, yet students’ mental health cries have been publicly ignored, just like our petitions and protests for reduced tuition fees. How are unemployed students supposed to support themselves when they can’t receive furlough payments? But more importantly, who wants to fight for a minimum wage job that makes you feel expendable?

The pandemic has allowed valid criticism of the job market and how it is controlled. Why would anyone want to dedicate their time to an environment that doesn’t prioritise their employees’ health? Why are employers still allowed to ask invasive health questions or dismiss mental health problems? If you think I’m exaggerating, ask any young person if they’ve ever been afraid to take a sick day out of fear of getting fired.

### Yasmin

As someone who is autoimmune and suffers with an invisible disability—Ulcerative Colitis (UC), it was inevitable that I’d need to self-isolate and log off from the offline world. Sorting a day out with friends has always been a test of endurance, from finding places to eat that are UC friendly to obsessing where the nearest WC may be. Nonetheless, when I received my shielding letter advising me to “get fresh air by opening a window”, I was devastated. I was not aware that isolation would be a challenging experience for those deemed ‘Clinically Extremely Vulnerable’ and in their final year of university. Days of vomiting and cramps were an evil game of porcelain thrones, and the inflammation and dehydration left me weak in my bones. These laborious symptoms quickly escalated, and within the first two weeks of the academic year, I suffered an acute flare-up of my UC and was admitted to hospital.

I have been on–off shielding since April 2020 and whilst it has been difficult seeing nothing but four walls and suffering a flare-up, this was the least of my worries. I was troubled that my personal learning plan was not implemented during the academic year. While universities provided information on how to “stay safe” in a pandemic, there was scant information about how to “study in a pandemic when you’re ill”. Fortunately, I was able to design my own learning plan. I made sure tutors were aware that I was suffering a flare-up and that I would require extensions, I developed small deadlines to manage workload, and arranged one-to-one meetings.

As the eldest daughter in a South Asian family, I am responsible for multiple tasks such as household duties, being the mediator in familial affairs, and supporting family members’ well-being (whilst managing my own). Carrying these responsibilities intensified while shielding. I found it difficult to separate my social, home and study life. It came to a point where all aspects were under one roof. To overcome this, I identified the benefits of shielding, such as enjoying failed attempts at baking banana bread with my siblings, becoming cautious about my well-being, and bettering my relationship with Islam. There’s a particular verse in the Quran that I took comfort by, “Verily with every hardship comes ease”.

Whilst I have been vaccinated, I worry about feeling pressured to re-enter the world. I believe we should think about this period as a ‘maintenance break’ for our overactive brains, helping us improve on wellness, and enjoying the insignificant things like taking short walks, having a movie marathon, or making enough pasta to feed your whole postcode.

### Katerina

Having a false negative COVID-19 test made me feel depersonalised by the health system and my university. Looking back, there’s a shadow of imposter syndrome and feelings of neglect over my experience. My negative test meant my symptoms were up for debate and, often, denied.

I experienced draining COVID symptoms for three months in 2020. It began with high fever and sharp pain in my lungs. I followed government guidance and got a test within one to five days of experiencing symptoms, which came back negative. As you can imagine, I was relieved. Nobody wants a potentially deadly virus. But as I continued to have a high fever and feel unwell, I naturally began looking for explanations as to why I wasn’t recovering and if I could safely return to university.

Wondering why my condition wasn’t improving two weeks later, I called my doctor. He laughed off my question about the possibility of a false negative test as “highly unlikely”. For months afterwards, I questioned every moment of that phone call with the doctors. I should have been more assertive about my symptoms. I should have insisted he tell me the statistics on false positives. What if he advised me to get a test that day and it came back positive? These thoughts haunted me whenever someone didn’t believe I was sick.

After another week of fever, I called the doctors again. This time I was told my symptoms were in line with other COVID patients and that I should be tested immediately. However, the five-day window in which testing can pick up the virus had now been long closed. Predictably, I had another negative test. By this time, I had developed extreme exhaustion, which made my arms and legs feel heavy, like paralysis. A positive test was all I wanted to understand what I was going through. I wanted someone to give me an undisputable reason to stop everything and rest.

Instead of resting, I did what I felt like I was supposed to do. I carried on working. I began doubting my exhaustion because my negative test was supposed to be a sign of my health. I regularly emailed my tutors updating them about my poor condition. I completed my assignments despite being unable to sit up straight for more than an hour. I did it because there was no other option. The only time faculty checked up on me was months later when they noticed a drop in my attendance. I felt like a number in a system, not a person who was struggling through illness.

### Nasira

I made a choice in 2020 to return to South Africa, where my parents live, because of COVID-19. I hadn’t seen them for almost a year, university was online, and it was coming up to the Christmas break. Leaving the UK meant leaving my independence, and leaving my two sisters and their families, but it also meant having the opportunity to be with my parents, my eldest sister, her family and my significant other. It was now or never. The UK’s borders were open, and I knew if I didn’t leave, I’d be trapped. In fact, the day after I left, the PM shut all borders in preparation for the second wave. I embraced the change because change is inevitable. However, what I didn’t realise at the time was how many challenges I would face, and how much my life was going to be affected by the choice I made.

On the 1st of January, my dad and sister tested positive for COVID-19 and naturally the rest of us followed suit. With exams and deadlines approaching, I made the crippling decision to get an extension. Though my symptoms improved, my dad got progressively worse, and after ten days at home he was admitted to hospital in a life-threatening condition. Next came the decision to put him on a non-invasive ventilator. In our lounge, the doctor sat us down and told us, “I do not know how long he has left”.

Four arduous months passed, and I had re-defined heartbreak. From watching my dad present my 21st birthday speech to feeling his pain as he battled to merely lift his head off the bed; I was paralyzed with the notion “Life is precious, life is unexpected, to live, is to love life.” Although the hardships of these past months have been the chorus to my depiction of life, I’ve confronted my most daunting demons and I can finally see the light.

Knowing pain also means knowing love. The beauty in pain means you were fortunate enough to experience rare, pure love. I chose to have faith in my dad’s recovery, despite the prognosis. I kept working because I was positive when my dad got better, he’d want to see my second year of university completed. If I was asked to go back in time and make a different choice, I would 100% say no. In my religion we believe with hardship comes ease, and truly I cannot relate to that more than I do now. He’s starting to walk again, and in many ways, so am I.

### Jessica

Defining a criterion for ‘the worst year’ is hard. Was it 2020? For some it was 1939, the first year of World War Two. The Mayans believed it would be 2012. For me, it was 2018. This was the year I started university, the year I started living independently. This should have been exciting, right? Instead, it become a trigger for my mental health struggles.

My battle with depression, anxiety, and OCD began in 2008 when my family immigrated. Through a series of unfortunate events and pressures to be some version of “successful”, I reached a breaking point in 2018. I had to choose, either manage these illnesses or surrender to them. I chose to manage them, and this became fundamental to my life. There wasn’t a single resource that facilitated the management of my struggles, most of the work required to dig myself out of the hopelessness that comes with these afflictions could only be done by me. One of the most important lessons I learned was to find the silver lining amidst the darkness. A sentiment I believe many can relate to from their experience during the pandemic.

The impact of COVID-19 has been massive. It has amplified all social crises: austerity, the environmental crisis, racial and gender inequality, immigration, and Brexit. With my family in South Africa, I was having to live through different versions of the pandemic, having to face the exhausting reality that I was too far away, to help or say goodbye. However, the biggest impact of the pandemic for me was the precarious sense that, if needed, any call for help would be put on hold.

The truth is, there is no how-to guide for life. Recognising that many people have already had their worst year is important. Many have been dealing with issues before this pandemic, and they will continue to battle with them over the course of their lives. It is imperative to remember, that, whatever the year, people are struggling. But we should strive to find those moments of happiness. That moment might be big, like getting your best result on an assignment, or small, like the great smell of your coffee in the morning. At least if that’s learnt from my experience of 2018 to 2020, next time we are faced with a crisis, more people will feel hopeful in their survival.

### Charlotte

I have dreamt about studying at Lancaster University for as long as I can remember, but the safety of education suddenly felt lost in the pandemic. Though it would have been easy to give up, I did not want to let my dreams slip away. My experience since the pandemic started has been a series of uncertainties. The safety of my plans for the future felt jeopardised. Since March 2020 I have been furloughed, then at risk of redundancy, then safe, then made redundant, and then unemployed. I have also been the support mechanism for my family members who have had COVID-19. Those two things were the most significant disruptions. Or, they were until I took control of my mental health. I am on an ever-developing path of learning what works for my mental health. I have decided that I will do everything I can to achieve my goals. I do not want to focus on negative experiences. I have been able to turn those experiences into opportunities.

I have a newfound appreciation for the small things. I have found new walking routes in the place I have lived in for eighteen years, by exploring the landscape with my dog and my horse. We have had a year of adventures and creating memories, ones that are more than pictures. I have helped my friend through the grief of losing her dad. The days out with our horses showed us that he was still with her, just in different ways. We have focussed on the little things that make the big things.

I am on a journey of improving my mental health. I am happy with myself at university. I am starting my Masters degree in English Literary Studies at Lancaster University in October 2021. I am a published author. Though I had previously avoided creative writing for fear of being exposed, during the pandemic I learned to embrace it and find ways to communicate my experience. I have written about the beauty of being in nature. I have opened up. And, I have a new job. All of these achievements have only been attainable because of everything I have been through. It is through self-growth and development that I have enhanced myself and am on my own route that makes me happy.

## Discussion

Our individual reflections on the pandemic demonstrate how societal, political and institutional responses have exacerbated existing precarity and intersectional inequalities experienced by young people. Reflecting emerging research on youth experiences of the pandemic (e.g. Bengtsson et al 2021; Burns et al 2020; Cook et al 2021), education, employment, and mental health surfaced as key concerns. Across these interrelated aspects of experience, there is a shared sense of being let down, undervalued, and ignored by institutions and structures. We argue that this perceived neglect, as well as the physical isolation imposed during the pandemic via lockdowns and social distancing, has heightened our experiences of neoliberal individualisation and responsibilisation (Farrugia 2011; Kelly 2001). This is reflected in our narration of the pandemic as a personal journey in which we have sought our own solutions to the challenges faced. At the same time, our accounts are inflected with a sense of hope, grounded in the precarious present, which is oriented to social solidarity and individual and collective transformation for a more secure future.

In the context of higher education, unequal access to and precarious progression through university has become more acute. Our accounts call into question universities’ capacity to respond effectively and empathetically to students who are struggling to manage the generalised anxieties and uncertainties of pandemic life, and more particularly, those with both pre-existing and COVID-related illnesses. Living with a disability that renders her clinically vulnerable to COVID-19, Yasmin’s account suggests a lack of faith in the system to provide adequate support and points to the extra burden placed on disabled students during the pandemic. Similarly, Katerina expressed a sense of invisibility, of being ‘a number in a system’ as she struggled with the effects of illness. Reflecting more broadly on higher education access and progression, Rhiannon drew on her own experience as a first-generation student from a ‘deprived’ area to highlight how responses to COVID-19 have further capped aspirations for young people who share her background. Our reflections also reveal that precarity in the arena of paid work—already a significant issue for young people (Macdonald and Giazitzoglu 2019)—was exacerbated by the pandemic. Echoing Cook et al.’s findings (2021), the impact of precarious un/employment is felt most profoundly by those who lack access to other financial resources. This is the case for Isabel, who, as ‘a first-generation student from a working-class Caribbean family’, also faces multiple intersecting barriers to secure work. Her account of mental and physical exhaustion, of fear and grief, points to the toll of the youth labour market, pre- and during the pandemic, that extends far beyond financial precarity.

The turn to considering mental health in different experiential contexts (work, education, life as a transnational citizen) recurs through our reflections. Again, we contend that the conditions contributing to mental ill-health for young people pre-existed and were exacerbated by the pandemic. Our reflections describe the effect of precarious lives in a variety of ways, including worry, stress, the need to rest ‘tired’ brains, self-doubt, depression, exhaustion, imposter syndrome, and feelings of being neglected and isolated. Furthermore, Isabel reveals the fear that young people can experience about disclosing mental health struggles, and Jessica alludes to the strain on services: ‘the precarious sense that if needed, any call for help would be put “on hold”’. Again, the word precarious emerges here to define a pre-existing crisis for young people.

Through all our reflections, we identify highly individualised responses to the pandemic. We contend that this focus on the individual emerges from a wider ‘neoliberal social imaginary’ that dominated before the pandemic (Evans and Sewell 2013: 37). From Yasmin finding her own solutions to studying whilst shielding to Charlotte’s determination to take control of her own mental health, our reflections reveal the intensification of individualised forms of subjectivity that go hand in hand with increasing structural insecurity (Beck 1992). Our reflections suggest that the impetus for young people to respond as individuals to social crises and structural inequalities has been exacerbated by the physical and social isolation imposed by COVID-19. For some, such as Katerina and Nasira, this has caused anxiety and resulted in a sense of personal insufficiency as they found themselves unable to successfully navigate the multiple challenges they were facing (Farrugia 2011). For others, however, such as Charlotte, the pandemic was experienced as an opportunity for ‘self-growth and development’. Charlotte’s account evokes the figure of the ‘entrepreneurial self’ (Kelly 2007): a self-managing, individual constructed in response to crisis and instability. Charlotte’s positionality, which affords her relative security, supports a biographical account in which she ‘takes charge’ of her mental health and uses the time and space afforded by the pandemic to pursue her goals (Cook et al 2021). However, running through our reflections, we also find that this self-managing, responsible individual is at risk of being swamped and exploited by ‘mentally and physically exhausting’ systems and institutions, from the medical establishment to the job market. Troublingly, we all felt compelled to conduct ourselves as though it was ‘business as usual’, even in the midst of individual and family crises. This is encapsulated by Nasira, who, having relocated to South Africa, suffering from COVID-19, and with her father critically ill, experienced asking for an extension on university assignments not as a reasonable response to extreme circumstances, but rather as a ‘crippling decision’.

Writing these reflections has made clear that young people are seeking to imbue the pandemic with meaning. We recognise a tendency to characterise our experiences of the pandemic through the dyadic imagery of finding ‘light’ in the ‘darkness’, and through the Islamic idea of ease through hardship, which we attribute to this desire to find meaning. In this way, our reflections echo a wider societal imaginary that holds the pandemic as both a crisis and an opportunity. Considering the evidence that the pandemic has reinvigorated mutual aid in society; for example, Springer (2020) describes it as an ‘interregnum’, a moment of uncertainty that contains possibility and hope. Our language is equally figurative and perhaps more dramatic: Jessica evokes images of ‘battle’ and ‘surrender’, of navigating the ‘twists and turns’ of the pandemic, to end in a place of hopefulness. Nasira describes her experience of ‘confronting demons’ but suggests that experiencing pain allowed her to know beauty, also. These are not so much sanguine responses to the pandemic, capitulations to the neoliberal ideology of resilience, as accounts that imbue narrative meaning to the crises we have navigated. In this sense, our experiences differ from the sense of the quiet interregnum of the pandemic as it is often portrayed in the popular media: a period of quiet working from home; and an opportunity to re-assess family life and priorities—at least for financially secure adults. Our accounts are altogether more visceral, but we have sought to identify legacies for personal development and social change in our negative experiences of the pandemic, whether this be in Rhiannon’s determination to make a difference through dedicating herself to education or Charlotte’s evocation of a ‘journey’ of personal growth.

Emerging out of the challenges of the pandemic, and our attempts to find meaning in them, is a sense of what we have termed *precarious hope*: hope that emerges through—rather than despite—precarity; through the instability and uncertainty that has ruptured existing practices and structures and thus rendered the future mutable. At the same time, it is a hope that is itself precarious, vulnerable to the vicissitudes of contemporary youth experiences and to the forces that structure them. There is an extent to which this hope is utilitarian, a strategy for coping in extreme circumstances; however, as Cook writes (2018: 112), such hope is ‘not necessarily symptomatic of denial or naivety’. Rather, following Freire (2014), we argue that what we are experiencing is ‘critical hope’: hope that is grounded in the real possibilities for transformative change emerging through contemporary conditions of precarity, but that is also cognisant of the socio-political constraints on its realisation (Millar 2017; Tsing 2015). This precarious hope emerges most clearly in Isabel’s observation that the pandemic has opened up space for critically engaging with the youth job market, and her hope, also expressed by Rhiannon in relation to education, that the societal empathy fostered through the pandemic might be brought to bear on tackling structural inequalities. It is also visible in Yasmin and Jessica’s hopes that the pandemic has taught us to value and nurture our well-being so that we are better equipped to weather the precarious future.

## Conclusion

The precarious hope that emerges throughout this article is, as Freire (2014) argues, a critical pre-condition for action. We do not, however, believe that such action can or should be the sole responsibility of young people. As our narratives attest, young people’s experiences of the COVID-19 pandemic have been characterised by the ignoring and silencing of our voices in political and institutional settings, even as our responsibility for shaping our own biographies has been amplified. Addressing the challenges that lie ahead requires not only collective action but also cross-generational dialogue, understanding, and solidarity. Crucially, it requires the inclusion of youth voices in conversations about the present and the future. This article, co-produced through critical and caring dialogue among undergraduate students and older adult academics, is our contribution to this conversation.
